# Phenotypic Stability of *Staphylococcus aureus* Small Colony Variants (SCV) Isolates from Cystic Fibrosis (CF) Patients

**DOI:** 10.3390/ijerph16111940

**Published:** 2019-05-31

**Authors:** Clemens Kittinger, Daniela Toplitsch, Bettina Folli, Lilian Masoud Landgraf, Gernot Zarfel

**Affiliations:** Diagnostic and Research Center for Molecular BioMedicine, Medical University of Graz, 8010 Graz, Austria; clemens.kittinger@medunigraz.at (C.K.); daniela.toplitsch@medunigraz.at (D.T.); bettina.folli@medunigraz.at (B.F.); lilian.masoud@medunigraz.at (L.M.L.)

**Keywords:** cystic fibrosis, lungs infection, thyA, revertant

## Abstract

One of the most interesting features of *Staphylococcus aureus* is its ability to switch to a small colony variant (SCV). This switch allows the pathogen to survive periods of antibiotic treatment or pressure from the immune system of the host and further enables it to start the infection once again after the environmental stress declines. However, so far only little is known about this reversion back to the more virulent wild type phenotype. Therefore, this study aimed to analyze the frequency of reversion to the wild type phenotype of thymidine auxotroph *S. aureus* SCV isolates (TD-SCVs) obtained from patients with cystic fibrosis (CF). With the use of single cell starting cultures, the occurrence of the thymidine prototroph revertants was monitored. The underlying mutational cause of the SCVs and subsequent revertants were analyzed by sequencing the gene coding for thymidylate synthase (ThyA), whose mutations are known to produce thymidine auxotroph *S. aureus* SCV. In our study, the underlying mutational cause for the switch to the TD-SCV phenotype was primarily point mutations. Out of twelve isolates, seven isolates showed an occurrence of revertants with a frequency ranging from 90.06% to 0.16%. This high variability in the frequency of reversion to the wild type was not expected. However, this variability in the frequency of reversion may also be the key to successful re-infection of the host. Sometimes quick reversion to the wild type proves necessary for survival, whereas other times, staying hidden for a bit longer leads to success in re-colonization of the host.

## 1. Introduction

During the process of infecting a new host, *Staphylococcus aureus* undergoes various methods of fast adaptation and differentiation in the host environment. In the lung of patients with cystic fibrosis, *S. aureus* has to deal with pressure from the immune system of the host, antibiotics, interspecies competition, hypoxia and starvation. One form of adaptation to these challenging environmental conditions is switching to a small colony variant (SCV) [1,2,3,4].

The occurrence of a thymidine-dependent auxotrophy is most common in *S. aureus* SCVs that are isolated from clinical samples, e.g., patients with cystic fibrosis (CF) [1,5].

The thymidine dependency of these SCVs is genetically based on random mutations in *thyA*, the gene that encodes for the thymidylate synthase [2] The mutations can be located anywhere in the gene and can either be point mutations, insertions or deletions. The acquisition of such mutations has been shown to be induced by the frequent use of the antibiotic trimethoprim-sulfamethoxazole (TMP-SXT) for the management of chronic lung infection in CF patients [6]. Adaptation during the colonization and/or infection process of *S. aureus* seems to be guided not only by regulatory processes, but also by the occurrence and selection of changes in the DNA, which leads to the accumulation of various mutations throughout their entire genome [2,7]. 

*S. aureus* SCVs are characterized as a phenotype with impaired slow growth and repression of different virulence factors, furthermore, they exhibit increased cellular adhesion, invasion, and intracellular persistence capacity. This persistence in combination with the ability of (some) SCVs to revert to the wild type phenotype, leads to the special clinical impact of *S. aureus* SCV [6,8]. SCVs can be a significant part of the total *S. aureus* population in the lung of CF patients and their appearance is associated with a worse clinical outcome [9]. Recent studies observe the difficulty in obtaining an estimate of SCV reversion events in the patient. In addition, there are only a few studies that experimentally consider the genetics of reversion, in particular, the possibilities of whether and how such reversions take place. However, as of yet, no methods to quantify these events have been developed until now [10,11].

The aim of the study was to establish a reliable test for the estimation of the frequency of revertant occurrence of clinical *S. aureus* SCV isolates. In addition, this test should be used to evaluate the difference in the reversion frequency of clinical *S. aureus* SCV isolates.

## 2. Material and Methods

### 2.1. Bacterial Strains

The origin of clinical isolates and characterization of isolates were described previously [3].

In total, twelve thymidine-dependent clinical *S. aureus* SCV isolates and eleven *S. aureus* wild type isolates, with the corresponding spa type and the same origin, were included in this study. For SCV 3, there was no wild type isolate available (Table 1).

As a control strain, *Staphylococcus aureus* subsp. *aureus* Rosenbach 1884 (DSMZ-799) from the Leibnitz Institute DSMZ (German Collection of Microorganisms and Cell Cultures) was used.

### 2.2. Spa Typing

Spa typing is based on sequencing the polymorphic X-region of the protein A gene (*spa*). This is a well-established technique for *S. aureus* and the results can be compared with databases (http://spaserver.ridom.de/). The determined spa types can be assigned to distinct groups with known origin and similar characteristics.

### 2.3. thyA Sequencing

For sequencing of the *thyA* gene, *thyA* was amplified using primers thyA8F 5′-CATATTCCTAAAATGAGTGA-3′ and thyA6R 5′-AACGTTTAACACAAAAAAGTACTTT-3′, which included the start codon, as well as the stop codon of *thyA*. The following thermal cycling conditions were used: 95 °C for 10 min, (95° C for 45 s, 52 °C for 45 s, 72 °C for one min) for 35 cycles, 72 °C for 10 min and hold at 4 °C. The PCR was performed using the Q5^®^ High-Fidelity DNA Polymerase 2000 U/mL with the Q5^®^ High-Fidelity 2X Master Mix (all by New England Biolabs^®^ Inc., Frankfurt, Germany).

The sequences were regarded as wild type sequences if the protein sequence of ThyA was identical to the consensus sequence (NCBI Reference Sequence: WP_000934885.1) and/or with the ThyA sequence from the corresponding wild type isolate with the identical spa type. 

### 2.4. Auxotrophism Test

The auxotrophism of the SCV isolates was determined using the agar disc diffusion test according to Maduka-Ezeh et al. on Mueller–Hinton agar for thymidine [12].

### 2.5. Analysis of Reversion Frequency

SCV isolates were cultivated on Columbia Blood Agar Plates. From a single colony, 3 mL of 0.85% NaCl solution was inoculated and then diluted in 0.85% NaCl solution to reach an approximate concentration of one cell per 2 µL. Depending on the growth of the isolate, the dilutions used ranged from 1:250 to 1:1000. Of the dilution, 2 µL was added to 200 µL of LB media per well in a Honeycomb 100 microplate and incubated for 24 h at 37 °C with continuous shaking. The OD_600_ was measured every five minutes. The dilutions were deemed appropriate if no more than around 90% of the wells of the plate showed visible growth. Using the Bioscreen C system at OD_600_, the growth was tracked over the entire incubation period. The cultures with similar growth characteristics to the wild type were disregarded. Hence, wells with present revertants in the selected colony for inoculation were excluded from the calculation because the reversion already happened during growth on the agar plate. 

By visual inspection, each well showing *S. aureus* growth was counted for further calculations. Then, 2 µL of the culture suspension was dripped on Müller-Hinton (MH) agar plates and incubated for 24 h at 37 °C. The revertants growing on the Müller-Hinton agar plates were identified as *S. aureus* isolates using the species identification system by MALDI-TOF MS Axima™ Assurance System by Shimadzu (bioMérieux, Marcy-l’Etoile, France). To exclude possible contamination with other *S. aureus* strains, the clonality of revertants and their corresponding clinical SCV isolate was also confirmed via spa typing. The revertants were counted if both the identification with MALDI-TOF MS Axima™ Assurance System and the spa typing with the Ridom StaphType database excluded contamination. The LB broth contains thymidine, whereas Müller-Hinton agar plates do not contain thymidine. Thus only revertants with a functional thymidylate synthase can grow on Müller-Hinton agar plates, whereas TD-SCVs can only grow in LB broth and not on Müller-Hinton agar plates.

The calculation of the reversion frequency was performed by dividing the number of wells that could grow on MH agar plates by the number of wells showing growth in the Bioscreen C system.

## 3. Results

All *S. aureus* SCV isolates used in this study tested positive for thymidine dependency and showed no growth on MH agar plates or broth, but showed growth on LB agar plates and in LB.

Only one wild type isolate (corresponding to SCV-13) showed a different amino acid sequence than the consensus sequence, with an L214P mutation. This mutation was also present in SCV-13. Sequencing of the *thyA* gene from the twelve isolates revealed two isolates with wild type sequences (SCV-4 and SCV-13) and seven isolates with point mutations, which resulted in changes in the amino acid sequence or nonsense mutations (T51M in SCV-1, L214stop and D305G in SCV-2, L2stop in SCV-3, N92K in SCV-6, P303R in SCV-8, Q124stop and F231Y in SCV-9, and T51A in SCV17). A further three SCV isolates presented with deletions in their *thyA* sequence (SCV-7, SCV-10, and SCV-14) (Table 1). 

In the revertant test, five SCVs were unable to revert back to the wild type phenotype, including the three isolates with deletion mutations (SCV-2, SCV-7, SCV-10) as well as SCV-6 and SCV-13.

SCV-17 revealed the highest reversion frequency with 90.06%, followed by SCV-3 with 41.63% and SCV-9 with 6.97%. All other isolates showed a frequency lower than 1%.

The *thyA* sequence of the revertants revealed either the wild type sequence again or an introduction of new point mutations. One exception was SCV-17, here, all tested revertants showed the T51A mutation, which was already present in the SCV isolate SCV-17. Therefore, in this case, the genetic base of the SCV phenotype was not present in the *thyA* sequence.

In comparison, isolate SCV-1, also having a point mutation at position 51, only had revertants exhibiting the wild type sequence with a threonine residue at position 51.

Reversion to the wild type sequence occurred also in SCV-8 (three out of four revertants) and in SCV-9 (five out of six revertants). Both strains also showed one revertant with a compensational mutation, P303G for SCV-8 and Q124K for SCV-9, which restored the ability to grow on MH agar. Only one revertant with a compensational mutation could be detected for SCV-2, with an L214Q mutation and SCV-3, with four L2Q and one L2W. 

Second point mutations of SCV-2 and SCV-9 were also present in all revertants (Table 2), concluding that this mutation does not lead to a thymidine-dependent auxotrophic phenotype.

## 4. Discussion

The phenotypical switch of SCV back to the wild type seems to play a major role in relapses of seemingly defeated infections. SCVs with a stable phenotype (produced by bigger genetic events, e.g., deletion or insertion) have shown to be a dead end, and their isolation from clinical samples may just be a symptom of the rapid and relentless adaptation processes that *S. aureus* undergoes during colonization and infection of the host. During the isolation process, SCVs with an unstable phenotype are more likely to be missed or lost in further conservation and analyzation steps, due to their switch back to the wild type [9,13,14,15]. 

This is also reflected in the tested CF isolates: Most of the analyzed TD-SCV isolates showed no or a very low frequency of reversion to a thymidine prototrophic phenotype. The isolate collection in our study has obvious limitations due to the low number of isolates (twelve TD-SCVs), however, it includes two isolates, which exhibited reversion to the wild type phenotype in more than 10% of a 24 h incubation period on Columbia Blood agar plates. It is to be expected that SCV in the patients also showed the full range of variants, from the stable phenotype to the very unstable. However, the difficult isolation, as well as the low number of isolates, does not allow for a discussion of their distribution.

Sequence analysis of *thyA* in the clinical TD-SCV isolates showed that the mutations occurred at various sites throughout the entire gene, which goes in line with findings from previous studies. The revisions of deletion mutants could not be observed in this study. This is probably mainly due to the fact that the deletions were quite large. Other studies could show that *S. aureus* SCVs, triggered by deletions (in this case, not in the *thyA* gene), can be reverted. The low number of isolates does not provide enough statistically analyzable data, but two strains showed mutation on position A51. In comparison with other previously published data, no concordance of mutation sites could be assessed in this study [5,6,16].

The divergence in the frequency of reversion in the isolates can be attributed to the genetic background of the different strains/isolates, although the sites within the genes make mutations and reversion more or less frequent.

All isolates in this study could be sub-cultured many times without antibiotic pressure present, so we concluded that influence from a priming environment causing the mutations should not be present. The quantification of the reversion frequency for clinical isolates is the big benefit of this study, even though it is a rather artificial setup, because this strategy can also be used for different substances (e.g., other antibiotics). Thus, one should be able to determine whether certain substances or certain circumstances cause an increase or decrease in reversion sequence in different clinical isolates.

We suggest that this is due to various other mutations that the TD-SCVs have acquired in their genome, which may also have an impact on their ability to revert back to the wild type phenotype [4,17,18]. Such mutations probably occur because of the hostile environment *S. aureus* encounters in the host lung, which in combination with the high basal mutation rate that some *S. aureus* strains show and the impaired DNA repair systems, might lead to the accumulation of various other mutations throughout their entire genome, not just in the *thyA* region.

## 5. Conclusions

In conclusion, this study shows the high variability in the ability of TD-SCVs to revert to the wild type phenotype. In this study, we were able to show that the reversion frequency not only depends on primary mutations in *thyA*—which leads to their thymidine dependency and thereby facilitates the resistance to the antibiotic trimethoprim-sulfamethoxazole—but also other factors, which have not been identified yet and need to be studied in further experiments, as the emergence of TD-SCVs in isolates from CF patients has a significant impact on clinical outcome.

## Figures and Tables

**Table 1:** Results of experiment to determine the reversion frequency of thymidine auxotroph *S. aureus* small colony variant (SCV) isolates from cystic fibrosis (CF) patients.

Strain	Spa Type	*thyA* Mutation	Wells with Growth	Wells with Revertants	Reversion Frequency
SCV-1	t12308	T51M	500	3	0.16%
SCV-2	t209	L214stop, D305G	751	7	0.93%
SCV-3	t012	L2stop	526	219	41.63%
SCV-4	t732	Wild type	639	1	0.16%
SCV-6	t355	N92K	608	0	0%
SCV-7	t8012	∆588-602	534	0	0%
SCV-8	t004	P303R	589	4	0.68%
SCV-9	t085	Q124Stop, F231Y	502	35	6.97%
SCV-10	t085	∆390-403	611	0	0%
SCV-13	t645	Wild type	588	0	0%
SCV-14	t015	∆588-603	526	0	0%
SCV-17	t9847	T51A	533	480	90.06%

**Table 2:** SCV and corresponding revertants with amino acid mutations in comparison to a reference sequence.

SCV Strain	Spa-Type	ThyA Mutation	Revertants	ThyA Mutation
SCV-1	t12308	T51M	Rev1/1	wild type sequence
			Rev1/2	wild type sequence
			Rev1/3	wild type sequence
SCV-2	t209	L214stop	Rev2/1	L214Q, D305G
			Rev2/2	L214Q, D305G
			Rev2/3	L214Q, D305G
			Rev2/4	L214Q, D305G
			Rev2/5	L214Q, D305G
SCV-3	t012	L2stop	Rev3/1	L2W
			Rev3/2	L2Q
			Rev3/3	L2Q
			Rev3/4	L2Q
			Rev3/5	L2Q
SCV-4	t732	wild type sequence	Rev4/1	wild type sequence
SCV-8	t004	P303R	Rev8/1	wild type sequence
			Rev8/2	P303G
			Rev8/3	wild type sequence
			Rev8/4	wild type sequence
SCV-9	t085	Q124Stop	Rev9/1	Q124K, F231Y
			Rev9/2	Q124Q (wild type sequence), F231Y
			Rev9/3	Q124Q (wild type sequence), F231Y
			Rev9/4	Q124Q (wild type sequence), F231Y
			Rev9/5	Q124Q (wild type sequence), F231Y
			Rev9/6	Q124Q (wild type sequence), F231Y
SCV-17	t9847	T51A	Rev17/1	T51A
			Rev17/2	T51A
			Rev17/3	T51A
			Rev17/4	T51A
			Rev17/5	T51A
